# What the Peace Programme Has Done

**Published:** 1921-09-17

**Authors:** 


### THE ROAD TO CO-ORDINATION.

### What the Peace Programme Has Done.

The first report of the Joint Council of the Order of St. John, which was recently issued, covers an active period of work from January 1920 to March 31, 1921, although it was in September of 1919 that the Joint Council was actually formed. The report falls naturally into two parts. The first concerns the work of the Joint War Committee for Service or ex-Service men. The second describes the new departments formed to carry out the Peace Programme of the two societies. The first, or post-war work, is financed by the Joint War Committee; for the Peace Programme money collected in various ways since the Armistice has to suffice. Though the attraction of war work is lacking, the departments dealing with tuberculosis and child welfare are said to be arousing much interest, enough, indeed, to support, and perhaps to expand, the programme already sketched. Sir Napier Burnett, as chief executive officer of the Joint Council, is in charge of the administration, and also of all plans for starting new branches of work. His general proposals for "a comprehensive system of preventive work" have received, we learn, in the main, the approval of the Ministry of Health, and such work is important not only for its avowed object, but also for the field which it opens to voluntary service.

It is impossible in the space at our disposal to examine this report at length, although the activities recorded constitute what is really a new ally to the voluntary system; and it is highly desirable to-day, when co-operation is so much discussed and so little understood, that every section of the voluntary system should become alive to the work of its associates that all may pull together and bring their activities into line. If we turn first to the Hospital Services department, we find recorded with just pride the Societies' recent and memorable survey of the voluntary civil hospitals outside London in England and Wales. It recorded, of course, in summary detail, the work done and the financial condition of each hospital during 1919. In the light of subsequent events it is interesting to remember that the ordinary income was analysed to show the percentage derived from workmen's contributions, patients' payments, and public services, *e.g.* the Ministry of Pensions, the War Office, and County and Borough Councils. On this followed a second survey, restricted to hospital finance, in order to show the effect of the war on income and expenditure from 1915 to 1919 inclusive. The report states that copies of the survey were supplied to the committee of the National Relief Fund and "proved of some assistance to that body in the distribution to the hospitals of the sum of £700,000."

On this followed the visits of the Chairman of the Joint Council and the Director of the Hospitals Department to a number of provincial centres, where conferences were held to discuss and explain the general position of the hospitals as revealed by the surveys mentioned. All the delegates to these conferences received a summary of the financial survey, and there can be little doubt that the result was to encourage and enforce a common view of the hospital position throughout the country. It has been the lack of such perspective which has hindered co-ordination hitherto. The old individualism of each hospital, now a hardy habit of mind, yields slowly, but it is yielding to a more statesmanlike and sympathetic point of view. The seeds thus sown germinated, and a voluminous correspondence and many personal interviews ensued at the Societies' headquarters, 19 Berkeley Street. Also symptomatic of the movement toward co-ordination was the establishment of the Regional Committees of the British Hospitals' Association, which last invited the Director of Hospital Services to give the inaugural address at three of the meetings of these committees, namely, at Birmingham, Manchester, and Leeds. The address at Birmingham has since been published and circulated by the British Hospitals Association.

In another direction also the Joint Council has exercised its influence. The Director of Hospital Services has been appointed to serve upon no fewer than ten committees, representing such diverse activities as the National Relief Fund, the Central Council for Child Welfare, the Central Committee for the Care of Cripples, the National Council for Combating Venereal Diseases, and the like. Of similar work for hostels and hospitals for ex-Service men we have no room to speak; but several special inquiries into the administration of particular institutions of this class have been undertaken. A general leavening of hospital activity by this officer may be said to have ensued. He has also addressed numerous local bodies on hospital administration, finance, the co-ordination of voluntary civil hospitals, and similar matters.

After reading this account of activity in the spheres of analysis and propaganda, we turn to the work accomplished by the department of Organisation and Appeals. It assisted in the appeal for "Our Day," 1920. From the report of the Joint Council Finance Committee we find that the arrangements made for an "Our Day" collection in July 1920, under which in London 10 per cent. and in the counties 75 per cent. was to be retained locally, produced a sum of £50,360, of which roughly one-third was retained by the counties and two-thirds by headquarters. The total was disappointingly small—much smaller than we ourselves had hoped. But now that the war is over the Red Cross can no longer, by the raising of a finger, receive a million: time for preparation was short, and industrial depression had begun. The "Our Day" collection to be held this year, for which all arrangements and propaganda are being directed at headquarters, will be a better index of the yield to be expected in peace-time. We do not mean that the proceeds will necessarily be greater than last year. We mean that conditions now resemble more closely normal peace conditions.

Among many interesting matters two more must receive mention. The first is the scheme, detailed in Appendix II., for the organisation of a County Joint Committee. The scheme has been adopted by one county, and its form serves as a useful working model. The second matter worth study is contained in Appendix III., where the work and organisation of medical supply depôts, of which a number is in existence, are set forth. These useful spots are still too little known. Once placed in a central and accessible position, they can be of great service to all who know how to use them rightly. Their stock, which runs from Bath-chairs to sputum-mugs and air-cushions, is mainly for hire, at a cost of anything from 2d. to 2s. a week. The conditions are the return or replacement of the article, and "to ensure proper use of the articles loaned it seems advisable to issue articles on hire on vouchers signed by the Medical Officer of Health, local doctors, district and village nurses, recognised officials of charitable or infirmary institutions," who should receive books of vouchers (see Appendix III.). A few articles like pneumonia jackets, lint, wool, and gauze are for sale at modest prices. Sick-room necessities are everywhere required, usually for short periods only. These medical-supply depôts exist to furnish them. The depôts cannot be too widely known, or too carefully distributed. A handy directory should be available. The depôts still badly need publicity. There would be many more of them if those already existing were better known. In all the above activities, is there not welcome evidence of an attempt to fill some of the gaps and bridge some of the channels which at present make our hospital system incomplete?

